# Supplementary material for: Assessing the Clinical Characteristics and Management of COVID-19 among Pediatric Patients in Ghana: Findings and Implications
Source: Antibiotics (Basel). 2023 Feb 1;12(2):283. doi: 10.3390/antibiotics12020283 (PMC9952352; doi:10.3390/antibiotics12020283)
Supplement: Supplementary file 1 [file antibiotics-12-00283-s001.zip › antibiotics-2142114-supplementary.pdf]

Supplementary Material – Table S1

<table>
<tr><th colspan="4">QUESTIONNAIRE FOR DATA COLLECTION</th></tr>
<tr><td>GENDER
o Male
o Female</td><td rowspan="2">RADIOLOGICAL FINDINGS
o Chest X ray
o Chest CT scan
o Others</td><td rowspan="2">PRESENTING COMPLAINTS
o Cough
o Sore throat
o Temperature > 38°C
o Headache
o Abdominal pain
o Vomiting
o O_2 saturation < 92%
o Diarrhea
o Others</td><td rowspan="2">COMPLICATIONS PRESENT
o COVID Pneumonia on Chest CT scan
o Acute Kidney Injury (AKI)
o Acute respiratory distress
o Acute Liver Injury
o Septic shock
o Venous and arterial thromboembolic events</td></tr>
<tr><td>AGE (years)</td></tr>
<tr><td rowspan="2">COMORBIDITIES
o Other infections
o Cardiovascular diseases
o Metabolic/endocrine diseases
o Neurologic conditions</td><td rowspan="2">OUTCOME
o Mortality
o Discharged home</td><td rowspan="2">LEVEL OF SEVERITY
o Mild/ Moderate
o Severe Pneumonia
o Severe Pneumonia with Acute Respiratory distress syndrome /Sepsis</td><td>YEAR OF ADMISSION (YEAR)</td></tr>
<tr><td>TRANSFER TO ICU
o Yes
o No</td></tr>
<tr><td colspan="4">ALL ANTIBIOTICS USED (for each drug indicate the name, dose, frequency, dosage form, route of administration and duration of regimen)
____
____</td></tr>
<tr><td colspan="4">Are the choice of antimicrobials used consistent with that in the STG 2020 for COVID-19 management according to level of severity of the disease diagnosed?
o Yes
o No</td></tr>
</table>
